# Across Clinical Profiles of Cardiorenal–Metabolic (CKM) Syndrome: A Phenotype-Driven Therapeutic Approach

**DOI:** 10.3390/biomedicines14061289

**Published:** 2026-06-05

**Authors:** Irene Carlino, Sonia Di Franco, Nicola Colalillo, Stefania Bisogno, Luigi Gennari, Alberto Palazzuoli

**Affiliations:** 1 Internal Medicine Unit, Department of Medical and Surgical Sciences, University of Siena, 53100 Siena, Italy; 2 Cardiovascular Diseases Unit, Cardio Thoracic and Vascular Department, Le Scotte Hospital Siena, 53100 Siena, Italypalazzuoli2@gmail.com (A.P.)

**Keywords:** CKM, cardiovascular kidney metabolic syndrome, obesity, cardiovascular disease, diabetic nephropathy, heart failure, type 2 diabetes

## Abstract

Cardiorenal–metabolic (CKM) syndrome has emerged as a unifying condition describing the interplay between metabolic dysfunction, chronic kidney disease, and cardiovascular disease. To address this concept, the American Heart Association, in a 2023 Presidential Advisory, presented an official statement to capture the transition from metabolic risk and subtle cardiorenal dysfunction to overt cardiovascular and renal disease. Although this framework provides a structured representation of disease burden and facilitates risk stratification, emerging evidence suggests that it is primarily focused on the progressive nature, whereas high-risk patients may experience sudden cardiac or renal events. While staging systems provide important tools for risk stratification, they remain primarily descriptive and do not adequately reflect the dynamic and non-linear interactions underlying disease progression. Importantly, patients exhibit substantial heterogeneity in dominant pathophysiological drivers, related to various baseline risk factors and primitive cardio–kidney disorders, that is not fully captured by stage-based classifications. Notably, we propose a phenotype-oriented approach to CKM syndrome based on the recognition that its clinical expression reflects heterogeneous and evolving pathophysiological mechanisms rather than a uniform disease trajectory. According to this strategy, the paradigm of management shifts from an evolutive concept to a more appropriate use of disease modifying agents with cross-organ effects. Sodium–glucose cotransporter-2 inhibitors (SGLT2i), glucagon-like peptide-1 receptor agonists (GLP-1a), and non-steroidal mineralocorticoid receptor antagonists (MRA) have demonstrated the ability to modulate key biological pathways across the cardiovascular, renal, and metabolic axes. Therefore, personalized management that identifies a specific strategy according to CKM phenotypes must be assessed.

## 1. Introduction

Cardiorenal metabolic (CKM) syndrome has emerged as a pathophysiological paradigm integrating the bidirectional and self-reinforcing interactions between metabolic dysfunction, chronic kidney disease (CKD), and cardiovascular disease (CVD) [1,2]. CKM has been conceptualized as a continuum spanning from early metabolic risk to overt cardiovascular disease, with the explicit goal of shifting clinical focus toward earlier prevention and coordinated care [3,4,5]. Accordingly, the CKM staging system (0–4) was developed to capture this longitudinal transition and to reflect an evolving understanding that cardiovascular risk is the result of maladaptive interactions among metabolic, renal, and vascular systems rather than the additive effect of isolated comorbidities [6,7].

Thus, CKM syndrome is not to be seen as mere coexistence of diabetes mellitus, obesity, hypertension, and CKD, but rather as a condition coming from several pathways related to various metabolic dysfunctions, leading to neurohormonal activation, endothelial injury, oxidative stress, and chronic low-grade inflammation [8,9,10]. These alterations contribute to progressive microvascular dysfunction, increased arterial stiffness, and progressive impairment of renal autoregulation, ultimately promoting both cardiovascular remodeling and renal injury [11]. The resulting bidirectional amplification between cardiac and renal dysfunction represents a central pathophysiological hallmark of CKM syndrome, and it has been associated with increased risks of heart failure, kidney disease progression, and cardiovascular mortality across diverse populations [12,13,14].

## 2. Methodology

This narrative review was designed to provide a phenotype-oriented synthesis of the cardiovascular–kidney–metabolic (CKM) syndrome spectrum. A structured literature search was performed to identify relevant studies, scientific statements, consensus documents, clinical guidelines, randomized controlled trials, meta-analyses, and mechanistic reviews addressing CKM syndrome, cardiorenal interactions, metabolic dysfunction, chronic kidney disease, heart failure, obesity, diabetes, inflammation, endothelial dysfunction, and phenotype-based therapeutic approaches.

The literature search was conducted using PubMed/MEDLINE and Google Scholar. The search included articles published from January 2000 to May 2026, with priority given to contemporary evidence. The following keywords were used for the search: “cardiovascular–kidney–metabolic syndrome” OR “CKM syndrome”, “cardiorenal metabolic syndrome”, “cardiorenal syndrome”, “chronic kidney disease” AND “cardiovascular disease”, “obesity” AND “heart failure with preserved ejection fraction”, “diabetic kidney disease”, “SGLT2 inhibitors”, “GLP-1 receptor agonists”, “mineralocorticoid receptor antagonists”, “finerenone”, “phenotypes”, “cluster analysis”, “machine learning”, “endothelial dysfunction”, “inflammation”, “vascular stiffness”, and “cardiorenal outcomes”.

Eligible studies were selected if they addressed one or more of the following domains: pathophysiology of CKM syndrome; AHA CKM staging and its clinical implications; limitations of stage-based classification; phenotype-based or cluster-based approaches to cardiometabolic, renal, or heart failure populations; and evidence supporting therapeutic strategies with cardiovascular, renal, or metabolic benefits. Particular attention was given to large randomized controlled trials, major cardiovascular and nephrology outcome trials, scientific statements, and high-quality reviews. Studies were excluded if they were not directly relevant to CKM syndrome or related cardiorenal–metabolic mechanisms, if they lacked sufficient clinical or mechanistic detail, or if they were available only as abstracts without adequate methodological information.

Because this article is a narrative review, no formal risk-of-bias assessment or quantitative meta-analysis was performed. The selected literature was then critically synthesized.

## 3. Current Appraisal and Evolution of CKM Syndrome

CKM syndrome is sustained by a network of interrelated mechanisms through which metabolic dysfunction progressively drives both cardiovascular and renal injury. Excessive and dysfunctional adiposity acts as an endocrine organ that promotes a chronic pro-inflammatory environment, disrupts adipokine signaling, and favors ectopic lipid accumulation across metabolically relevant tissues. Sustained metabolic stress contributes to the development of insulin resistance and endothelial dysfunction while increasing arterial stiffness and vascular tone, thereby accelerating both atherosclerotic processes and myocardial structural remodeling. Collaterally, the release of adipose tissue-derived inflammatory mediators, together with reduced adiponectin bioavailability, impairs nitric oxide-dependent vasodilation and amplifies systemic oxidative stress, ultimately leading to microvascular dysfunction and progressive impairment of renal autoregulatory capacity [15,16,17].

Hyperglycemia and insulin resistance further contribute to renal injury by disrupting intraglomerular hemodynamics, primarily through maladaptive afferent arteriolar vasodilation and impairment of tubuloglomerular feedback. Over time, sustained metabolic stress promotes structural damage at the glomerular level, leading to progressive nephron loss. These alterations create a permissive environment for the onset and progression of diabetic kidney disease (DKD) and contribute to the development of downstream cardiovascular complications [6,18,19].

Sustained activation of the renin–angiotensin–aldosterone system (RAAS) and downstream mineralocorticoid receptor signaling contributes to progressive cardiorenal injury by promoting sodium retention and blood pressure elevation while simultaneously driving structural remodeling within both renal and myocardial tissues. Beyond its hemodynamic effects, persistent mineralocorticoid receptor overactivation amplifies inflammatory and profibrotic pathways, facilitating extracellular matrix expansion and the development of interstitial fibrosis across the cardiorenal axis [12,20].

These profibrotic and inflammatory processes extend beyond the kidney to involve the myocardium, where sustained neurohormonal activation and oxidative stress promote structural remodeling through fibroblast activation and extracellular matrix expansion. The resulting alterations in myocardial compliance contribute to increased ventricular stiffness and impaired diastolic relaxation. In the context of CKD, additional systemic factors, including anemia, uremic toxin accumulation, and disturbances in mineral metabolism such as fibroblast growth factor-23 (FGF-23) excess, further accelerate myocardial remodeling, favoring the development of left ventricular hypertrophy (LVH) and concentric geometric adaptation [11,21,22,23].

Thrombo-inflammatory mechanisms further amplify this maladaptive crosstalk. E-selectin and P-selectin play a central role in endothelial and platelet activation. P-selectin is expressed on activated platelets and endothelial cells, whereas E-selectin is induced on endothelial cells by pro-inflammatory cytokines; both mediate leukocyte adhesion to the vascular wall, representing a key early step in inflammation and thrombosis [15,16,17]. In CKD, circulating levels of soluble P-selectin are significantly elevated and are associated with increased fibrinogen and von Willebrand factor levels, reflecting a prothrombotic state driven by endothelial dysfunction and platelet activation [18,19]. Beyond adhesion, selectins trigger intracellular signaling pathways that promote oxidative burst, degranulation, and the release of procoagulant and proinflammatory microparticles, thereby contributing to vascular injury and thrombosis [15]. Clinically, elevated E-selectin levels have been associated with acute kidney injury, incident CKD, and venous thromboembolism, further supporting their role as mediators of endothelial dysfunction and disease progression [17,18,19,20].

**Figure 1:**
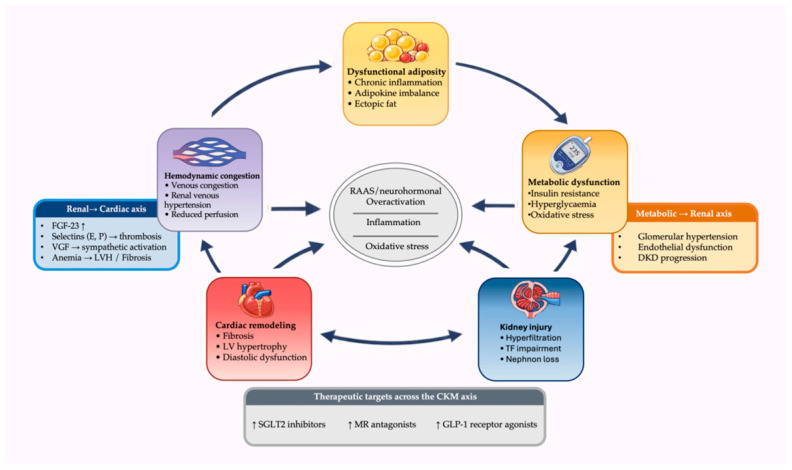
Integrated pathophysiology of cardiorenal–metabolic (CKM) syndrome.

In parallel, nerve growth factor inducible protein (VGF), a neuroendocrine granin protein expressed in central and peripheral neuroendocrine tissues, contributes to cardiorenal regulation through modulation of sympathetic activity and blood pressure homeostasis. Experimental models show that VGF deficiency is associated with increased catecholamine levels and hypertension, whereas the VGF-derived peptide TLQP-21 exerts antihypertensive effects [21,22]. These findings suggest that VGF may link neuroendocrine activation to cardiorenal dysfunction, complementing the hemodynamic and inflammatory pathways (Figure 1).

An additional gap lies in progressive framework deterioration, where cardiac and renal dysfunction should not be interpreted as parallel consequences of shared risk factors, but rather as mutually reinforcing components of a dynamic pathophysiological loop in which metabolic stress, inflammation, neurohormonal activation, and hemodynamic congestion act synergistically to drive progressive multiorgan injury. This bidirectional interaction is particularly evident in the cardiorenal syndrome, where alterations in cardiac function directly influence renal perfusion and venous pressures, while kidney dysfunction in turn amplifies neurohormonal activation, fluid retention, and systemic inflammation, thereby accelerating cardiovascular remodeling and disease progression [8,23,24,25].

In this framework, venous congestion and impaired forward flow represent complementary and often coexisting mechanisms of renal injury, with elevated central venous pressure emerging as a key determinant of worsening renal function independently of cardiac output [26,27]. Renal venous hypertension and increased intra-abdominal pressure in heart failure can directly impair glomerular filtration even in the absence of intrinsic kidney disease, contributing to cardiorenal syndrome through increased renal interstitial pressure and reduced renal perfusion [22,23,28,29].

Cardiorenal–metabolic (CKM) syndrome results from bidirectional interactions among dysfunctional adiposity, metabolic dysfunction, kidney injury, cardiac remodeling, and hemodynamic congestion. Shared mechanisms, including renin–angiotensin–aldosterone system (RAAS) activation, neurohormonal overdrive, inflammation, and oxidative stress, drive progressive multiorgan damage and sustain a self-amplifying vicious cycle. Additional contributors, such as endothelial dysfunction, anemia, thrombosis, and fibroblast growth factor-23 (FGF-23), further worsen disease progression. Major therapeutic targets acting across the CKM axis include sodium–glucose cotransporter-2 (SGLT2) inhibitors, mineralocorticoid receptor antagonists (MRAs), and glucagon-like peptide-1 receptor agonists (GLP-1 RAs).

## 4. Conceptual Limitations of Obesity-Related AHA CKM Definition

The AHA CKM staging system has several conceptual limitations that affect both its clinical applicability and theoretical coherence [1,30]. These limitations span disease heterogeneity, reliance on an adiposity-centered model, gaps in screening implementation, and an oversimplified representation of disease trajectories.

The rate and direction of progression across CKM stages vary considerably among individuals and are influenced by genetic predisposition, behavioral patterns, environmental exposures, and social determinants of health; however, the relative contribution and interaction of these factors remain poorly characterized [1,31].

**Figure 2:**
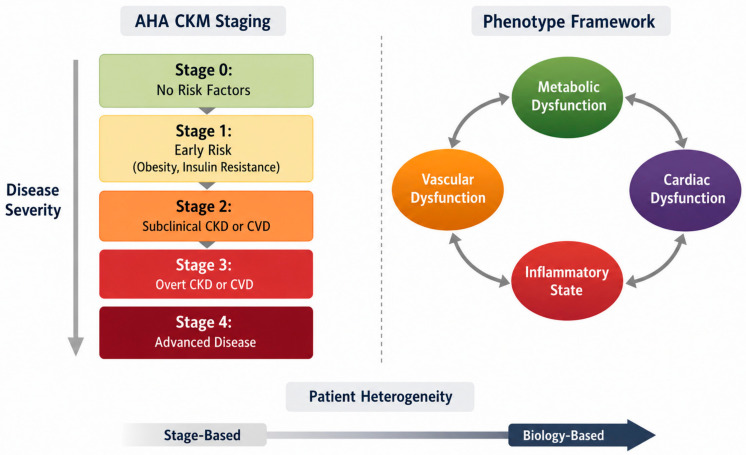
From stage-based classification to phenotype-oriented management in cardiorenal–metabolic (CKM) syndrome. The American Heart Association (AHA) CKM staging system stratifies disease severity from stage 0 (no risk factors) to stage 4 (advanced disease), reflecting progressive transition from early metabolic risk to overt chronic kidney disease (CKD) and/or cardiovascular disease (CVD). However, patients within similar stages may display substantial biological heterogeneity. A complementary phenotype framework identifies predominant drivers, including metabolic, vascular, cardiac, and inflammatory dysfunction.

The traditional nomenclature starts from an adiposity-centric conceptual model, particularly in Stage 1, where excess or dysfunctional adiposity is considered the primary initiating mechanism. This approach often fails to identify individuals at risk who develop metabolic dysfunction at normal body weight, as observed more frequently in non-White populations [1,32]. In addition, reliance on BMI and anthropometric measures introduces important limitations in accurately capturing adiposity, given their inability to distinguish fat distribution and metabolically unhealthy phenotypes [32,33,34,35]. Moreover, the model does not adequately account for patients who develop sudden cardiovascular events independently from metabolic pathways [36]. Individuals at intermediate stages may rapidly shift to advanced disease due to primary organ-specific insults [1,36,37]. A sudden acute cardiac event, whether related to plaque instability or other systemic mechanisms, can precipitate myocardial injury, leading to heart failure, consequent hemodynamic impairment and neurohormonal activation. For example, a young 45-year-old woman with Stage 2 CKM (hypertension and prediabetes, BMI 28) who develops spontaneous coronary artery dissection with acute myocardial infarction immediately shifts to Stage 4 CKM (clinical CVD with CKM risk factors) [36,37]. Similarly, primary renal insults such as acute kidney injury or rapidly progressive glomerular disease may trigger volume overload, RAAS activation, and cardiac hypertrophy, leading to acute cardiovascular deterioration. For example, a young 38-year-old woman with Stage 2 CKM (metabolic syndrome with mild hypertension) who develops rapidly progressive glomerulonephritis (RPGN) or secondary immune-complex nephropathy could experience severe acute kidney injury, with subsequent development of acute fluid retention and overload, and uremic cardiomyopathy, progressing toward Stage 4 CKM [38,39,40]. These trajectories underscore a key limitation of the CKM framework, which predominantly reflects metabolic-driven progression and does not fully account for acute cardiorenal interactions arising from primary cardiac or renal disease (Figure 2).

Although the framework is intended to be integrated with quantitative risk tools such as PREVENT, the applicability of these models appears to be stronger in the early stages of CKM syndrome. In contrast, their predictive performance progressively declines in acute clinical settings and advanced disease stages [36].

The absence of robust longitudinal studies incorporating competing-risk methodology confirms the limits in risk prediction and in clinical outcome prioritization, related to the heterogenicity of CKM components that may confer distinct risk burden not captured by a unidimensional staging model [1].

Important mechanistic knowledge gaps further limit the robustness of the model. While epidemiological associations between CKM components and cardiovascular outcomes are well established, the underlying biological pathways remain incompletely understood. Additionally, the transition from metabolic dysfunction to specific cardiovascular phenotypes, such as heart failure with preserved ejection fraction or atrial fibrillation, remains insufficiently characterized, as does the bidirectional relationship between cardiovascular disease and kidney dysfunction [1].

Collectively, these limitations highlight the need for a more nuanced, phenotype-oriented and multidimensional approach to CKM syndrome capable of integrating individual variability, mechanistic diversity, and competing risk trajectories to improve both risk stratification and therapeutic decision-making [1,41].

## 5. Possible CKM Phenotype Differentiation

Although current CKM staging provides a useful framework for stratifying disease severity, individuals in similar stages frequently display markedly different biological mechanisms and trajectories of organ involvement [1,42,43]. Recent clustering studies suggest that CKM syndrome comprises biologically distinct subgroups differing in risk factor burden, dominant organ involvement, and molecular signatures [44,45,46]. In a UK Biobank cluster analysis of 44,200 individuals with CKM stages 1–3, five distinct clusters emerged, including vascular–diabetes, vascular-dominant, metabolic-dominant, and cardiorenal profiles. Similarly, clustering analyses in broader CKM populations identified phenotypes ranging from low-risk groups to cardiorenal high-risk, liver high-risk, and cerebrovascular high-risk subgroups, with each exhibiting distinct plasma proteomic and genetic signatures [46,47]. Likewise, metabolomic profiling has demonstrated substantial heterogeneity even among individuals classified within the same CKM stage, with specific lipid signatures differentiating high- and low-risk trajectories [47]. Collectively, these findings support the concept that CKM syndrome should not be regarded as a uniform entity, but rather as a spectrum of interconnected phenotypes sharing common mechanistic pathways while differing in their dominant biological drivers [1,46,47].

Despite increasing recognition of this heterogeneity, current frameworks remain largely stage-oriented and may insufficiently capture the biological complexity observed in real-world disease progression. Recent data-driven approaches suggest that integrating cardiovascular, kidney, and metabolic domains may provide a more clinically meaningful strategy for patient stratification [48,49,50]. In a large retrospective analysis, Hsu et al. clustered CKM phenotypes according to the coexistence of cardiovascular, renal, and metabolic involvement, demonstrating that specific domain combinations carried distinct prognostic implications across multiple outcomes [51].

In accordance with current analysis and some epidemiological evidence, we propose six clinically recognizable CKM phenotypes integrating dominant cardiovascular, renal, and metabolic substrates.

**Figure 3:**
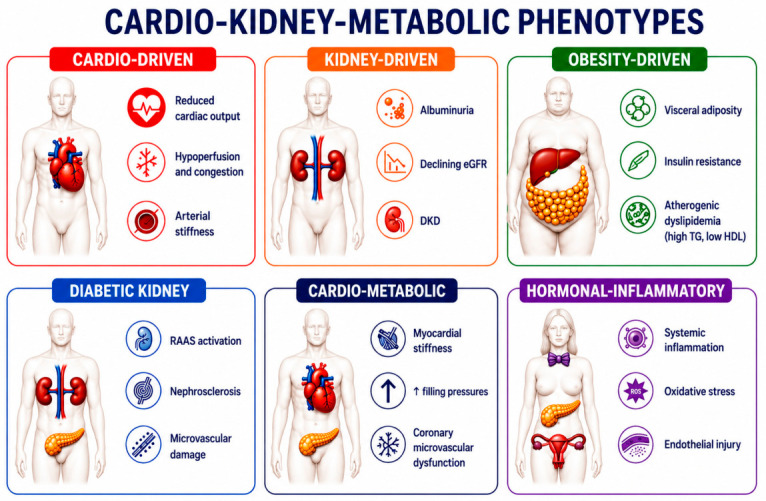
Proposed CKM phenotype classification. The schematic illustrates six biologically driven CKM phenotypes. Abbreviations: DKD, diabetic kidney disease; HDL, high-density lipoprotein; RAAS, renin–angiotensin–aldosterone system; TG, triglycerides.

The first phenotype may be defined as a cardio-driven profile, characterized by predominant involvement of the cardiovascular domain, including coronary artery disease, valve disease, cardiomyopathies, previous thrombotic events, and, frequently, heart failure with reduced ejection fraction (HFrEF) as the terminal manifestation of progressive myocardial injury. Within this subgroup, cardiovascular dysfunction represents the initiating biological substrate, whereas renal abnormalities frequently emerge as secondary consequences of reduced renal perfusion, venous congestion, and persistent neurohormonal activation (Figure 3).

A second phenotype corresponds to a kidney-driven profile, in which renal disease itself constitutes the principal pathological driver. This phenotype may encompass glomerular, tubulointerstitial, hereditary, or toxin-induced nephropathies, with CKD preceding and amplifying cardiovascular risk and heart failure development. Declining renal function, sodium retention, uremic inflammation, vascular calcification, and sustained neurohormonal activation create a systemic environment characterized by endothelial dysfunction, increased vascular stiffness, and multi-territorial calcification. Like observations reported by Hsu et al., kidney-domain involvement appears to identify individuals with particularly elevated mortality and adverse renal outcomes, emphasizing the kidney as a central amplifier of disease progression [51] (Figure 3).

A third profile may be identified as an obesity-driven phenotype, likely representing one of the most prevalent CKM presentations in industrialized societies. This subgroup is characterized by overweight or obesity-associated metabolic dysfunction, insulin resistance, dyslipidemia, and progression toward type 2 diabetes, often accompanied by early renal abnormalities such as albuminuria and mild reductions in glomerular filtration rate. At the cardiovascular level, visceral adiposity, metabolic stress, lipotoxicity, and endothelial dysfunction promote myocardial remodeling and impaired diastolic relaxation, progressively increasing susceptibility to cardiometabolic HFpEF. Sex-specific analyses suggest that men demonstrate greater prevalence of subclinical atherosclerosis and accelerated cardiovascular risk trajectories. This profile is coherent with “metabolic/renal dysfunction-dominant” clusters identified by machine-learning and EMR-based clustering models [52,53] (Figure 3).

A fourth phenotype may represent a hormonal inflammatory phenotype, more frequently observed in women and characterized by coexistence of metabolic dysfunction with a relatively greater prevalence of inflammatory and endocrine-immune-associated diseases. This cluster may promote systemic endothelial dysfunction capable of inducing myocardial and renal microvascular injury beyond purely hemodynamic mechanisms. Women exhibit greater propensity toward myocardial stiffening and subclinical heart failure manifestations despite having a lower atherosclerotic burden. This phenotype appears to be particularly relevant after menopause, when hormonal transitions contribute to altered adipose distribution and enhanced inflammatory activation [54,55] (Figure 3).

A fifth phenotype may be described as a cardio-metabolic profile, representing the paradigmatic expression of CKM syndrome. In this profile, obesity, hypertension, diabetes, atrial fibrillation, myocardial stiffness, elevated filling pressures, and coronary microvascular dysfunction coexist. Reduced renal reserve and increased susceptibility to congestion further amplify disease progression. Progression in this phenotype appears to be sustained by the integrated interaction among adipose inflammation, microvascular dysfunction, impaired ventricular relaxation, and fluid redistribution (Figure 3).

Finally, a sixth phenotype may correspond to a diabetic kidney profile, representing the classical renal–metabolic trajectory observed in diabetic kidney disease. This phenotype is characterized by persistent albuminuria, progressive decline in eGFR, type 2 diabetes, and a markedly elevated risk of heart failure and atherosclerotic cardiovascular events. In this context, the kidney acts as the principal disease amplifier through sodium retention, chronic inflammation, vascular calcification, neurohormonal activation, and accumulation of uremic toxins, accelerating myocardial remodeling and systemic vascular injury. Consequently, renal pathology becomes a major determinant of cardiovascular deterioration rather than merely a downstream consequence of metabolic disease [56,57,58,59,60] (Figure 3).

The phenotypes proposed in the present review were not derived from a formal Delphi consensus process, de novo machine-learning analyses, or direct observations from a single clinical cohort. Rather, they emerged from a narrative synthesis of available evidence integrating data-driven clustering studies, domain-based CKM classifications, sex-specific biological evidence, and mechanistic literature describing cardiovascular, renal, and metabolic interactions. Accordingly, the proposed framework should be considered a hypothesis-generating and mechanistically informed construct designed to translate current evidence into clinically recognizable profiles rather than a classification derived solely from personal clinical experience. The organization of these phenotypes reflects biological plausibility and translational applicability, with the aim of facilitating future phenotype-based approaches to CKM syndrome. Future longitudinal registries incorporating imaging biomarkers, kidney metrics, metabolic signatures, and machine-learning-based clustering approaches may validate these profiles and enable more precise identification of treatable traits, ultimately facilitating phenotype-guided precision medicine in CKM syndrome.

## 6. Diagnostic Criteria for CKM Phenotype Differentiation

**Figure 4:**
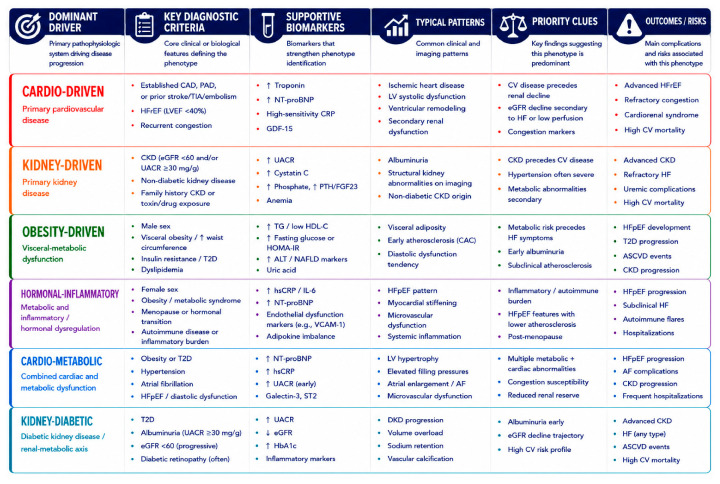
Proposed CKM phenotype diagnostic criteria for phenotype assignment.

Although these six CKM phenotypes provide a biologically plausible framework, their translation into routine clinical practice requires more explicit diagnostic criteria capable of differentiating overlapping disease trajectories and capturing the multidimensional nature of disease progression. Rather than relying exclusively on descriptive clinical characteristics, phenotype assignment may benefit from an integrated approach combining dominant pathophysiological drivers, core diagnostic criteria, supportive biomarkers, imaging patterns, and priority clinical clues. Such a strategy partially resembles the hierarchical logic adopted in AHA stage-based models while extending beyond disease severity by incorporating biological heterogeneity and mechanistic complexity (Figure 4).

From a practical perspective, phenotype assessment may follow a structured stepwise process aimed at identifying the mechanistic profile sustaining disease progression. The first step consists of confirming the presence of a CKM complex through evidence of cardiovascular disease or cardiovascular risk factors, kidney abnormalities (including reduced estimated glomerular filtration rate or albuminuria), and metabolic dysfunction such as obesity, insulin resistance, diabetes, or dyslipidemia. Once CKM involvement has been established, the second step focuses on identifying the prevailing disease mechanism or biological driver. In this phase, clinicians may determine whether the primary clinical burden is attributable to cardiac dysfunction, renal disease, or metabolic abnormalities.

When cardiac dysfunction predominates, indicators such as heart failure phenotype, reduced ejection fraction, elevated cardiac biomarkers, congestion-related manifestations, or cardiovascular structural abnormalities may orient toward a cardio-driven profile. Conversely, evidence of chronic kidney disease, albuminuria, progressive eGFR decline, or kidney-specific abnormalities may support a diabetic kidney phenotype. In patients with predominant metabolic burden, further characterization should distinguish between sex-specific and integrated metabolic patterns, recognizing differences in adiposity distribution, inflammatory burden, endocrine transition, and metabolic signatures.

The third step involves refinement and assignment of the phenotype through integration of supportive clinical and biological characteristics. Each phenotype may therefore be identified through a combination of variables reflecting the pathophysiological burden. For example, the cardio-driven phenotype may be supported by overt atherosclerotic disease, HFrEF, elevated cardiac biomarkers, and congestion-related renal impairment, whereas the kidney-driven profile may be recognized through CKD-specific features such as albuminuria, reduced eGFR, non-diabetic nephropathy, and biomarkers of mineral metabolism abnormalities. Similarly, sex-specific metabolic phenotypes may be differentiated through patterns of visceral adiposity, insulin resistance, inflammatory burden, endocrine status, and distinct biomarker signatures, including hsCRP, NT-proBNP, or markers of endothelial dysfunction. Imaging and functional parameters, including coronary artery calcium burden, diastolic dysfunction, filling pressures, atrial remodeling, microvascular abnormalities, or vascular stiffness, may further strengthen phenotype recognition.

**Figure 5:**
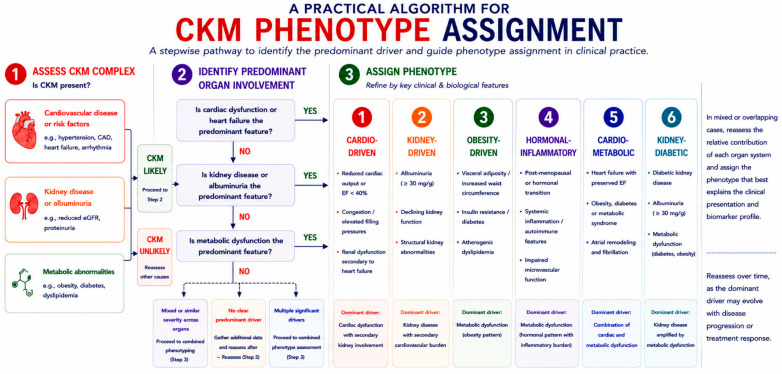
Practical algorithm for CKM phenotype assignment based on dominant biological drivers. Abbreviations: CKM, cardiovascular–kidney–metabolic; CAD, coronary artery disease; CKD, chronic kidney disease; eGFR, estimated glomerular filtration rate; EF, ejection fraction.

Importantly, phenotype assignment should not be interpreted as a rigid classification process but rather as a dominant-driver approach in which multiple biological domains coexist with varying degrees of contribution. In clinical practice, some individuals may exhibit mixed or overlapping phenotypes characterized by similar severity across organ systems or the simultaneous presence of multiple significant drivers. In these situations, the relative contribution of each organ system should be reassessed, and phenotype attribution should prioritize the biological substrate most strongly explaining the overall clinical presentation and biomarker profile. Longitudinal reassessment is also essential, as the principal disease axis may evolve over time in response to disease progression, therapeutic interventions, aging, or changing metabolic and inflammatory states (Figure 5).

Ultimately, such a multidimensional phenotype assessment framework may improve clinical applicability, facilitate reproducible patient stratification, and provide the foundation for future phenotype-guided precision medicine strategies in CKM syndrome.

This table gives a phenotype-oriented framework for the classification of patients across the cardiorenal–metabolic (CKM) spectrum, integrating dominant pathophysiological drivers with diagnostic, biological, and clinical characteristics. Abbreviations: AF, atrial fibrillation; ALT, alanine aminotransferase; ASCVD, atherosclerotic cardiovascular disease; CAC, coronary artery calcium; CAD, coronary artery disease; CKD, chronic kidney disease; CRP, C-reactive protein; CV, cardiovascular; DKD, diabetic kidney disease; eGFR, estimated glomerular filtration rate; FGF23, fibroblast growth factor-23; HF, heart failure; HFpEF, heart failure with preserved ejection fraction; HFrEF, heart failure with reduced ejection fraction; HbA1c, glycated hemoglobin; HDL-C, high-density lipoprotein cholesterol; HOMA-IR, homeostatic model assessment of insulin resistance; hsCRP, high-sensitivity C-reactive protein; IL-6, interleukin-6; LV, left ventricular; LVEF, left ventricular ejection fraction; NAFLD, non-alcoholic fatty liver disease; NT-proBNP, N-terminal pro-B-type natriuretic peptide; PAD, peripheral artery disease; PTH, parathyroid hormone; TG, triglycerides; T2D, type 2 diabetes; TIA, transient ischemic attack; UACR, urinary albumin-to-creatinine ratio; VCAM-1, vascular cell adhesion molecule-1.

## 7. Therapeutic Strategies Across CKM Phenotypes

In line with emerging evidence, the identification of clinically meaningful CKM phenotypes is shifting therapeutic decision-making from a purely stage-based paradigm toward a mechanism-oriented approach that targets the predominant biological drivers sustaining cardiorenal progression. This framework recognizes that hemodynamic congestion and neurohormonal activation, intrarenal microvascular and tubular stress, metabolic inflammation and fibrosis, and atherosclerotic vascular injury contribute heterogeneously across individuals, thereby defining distinct “therapeutic vulnerabilities” and priorities that may not be captured by disease stage alone [61].

Consequently, within a six-phenotype model, treatment can be conceptually aligned to dominant mechanisms rather than uniformly escalated across all CKM trajectories.

In the cardio-driven phenotype, progression is primarily sustained by hemodynamic impairment and maladaptive neurohormonal activation, with renal dysfunction often reflecting reduced forward flow, elevated venous pressures, and congestion-related kidney injury. Here, rapid implementation and optimization of guideline-directed HFrEF therapies remain foundational, and integrated management is essential to avoid inappropriate discontinuation of disease-modifying agents when modest early creatinine rises occur, which are frequently hemodynamic and not prognostically adverse [61]. Conventional evidence-based therapies remain fundamental in this phenotype. Renin–angiotensin system inhibitors, including angiotensin-converting enzyme inhibitors and angiotensin receptor blockers, together with beta-blockers and guideline-directed heart failure therapies, continue to represent cornerstone interventions in HFrEF and selected arrhythmic phenotypes. SGLT2 inhibitors added to guideline-directed therapy have consistently demonstrated reductions in hospitalization risk and improved cardiovascular outcomes across the heart failure spectrum. Importantly, their effects extend beyond glycemic control, including modulation of myocardial energetics, a reduction in congestion, and attenuation of renal injury [57,62,63]. Novel therapies should therefore be considered complementary rather than replacement strategies [64].

In contrast, the kidney-driven phenotype is dominated by primary renal parenchymal disease sustained by intrarenal hemodynamic and endothelial injury; in this setting, SGLT2 inhibition is central for restoring tubuloglomerular feedback and reducing intraglomerular stress, with robust guideline support across a broad eGFR range, including non-diabetic CKD, and should be integrated with etiology-directed nephroprotection, avoidance of nephrotoxins, RAAS blockade where indicated, and blood pressure optimization [65]. In parallel, interventions targeting CKD-associated metabolic abnormalities and vascular calcification may further reduce cardiovascular burden [66,67,68].

In the obesity-driven phenotype, characterized by visceral adiposity, insulin resistance, atherogenic dyslipidemia, and early vascular remodeling, persistent metabolic stress contributes to endothelial dysfunction, myocardial stiffening, and progressive renal injury. Lifestyle intervention remains the cornerstone of management, as structured programs combining dietary optimization, physical activity, and behavioral support improve insulin sensitivity and inflammatory burden [32,69]. Beyond lifestyle modification, glucagon-like peptide-1 receptor agonists (GLP-1RAs) have emerged as central therapeutic agents. In addition to promoting substantial and sustained weight reduction, GLP-1RAs have demonstrated cardiovascular protection across multiple randomized trials, reducing major adverse cardiovascular events independently of weight loss alone. More recently, the SELECT trial extended these benefits to patients with obesity and established cardiovascular disease without diabetes, reinforcing the concept of metabolic-targeted prevention [70,71,72,73]. Emerging evidence additionally suggests favorable renal effects through attenuation of albuminuria and slowing kidney function decline.

The hormonal inflammatory phenotype may represent a subgroup in which endocrine-immune and inflammatory drivers are comparatively more prominent, supporting an approach that combines cardiometabolic risk reduction with targeted management of endocrine/autoimmune contributors when present, and prioritizes therapies with proven benefits for weight, metabolic inflammation, and vascular risk in high-risk T2D/CKD/HF populations [12,74,75,76,77,78,79,80,81]. This phenotype may therefore particularly benefit from therapies capable of modulating systemic inflammation, metabolic dysfunction, and adipose tissue biology [29,63,82,83,84,85,86]. Beyond GLP-1RA-mediated metabolic effects, increasing attention has focused on sex-specific determinants of HFpEF and on the interaction between hormonal dysregulation and cardiometabolic remodeling [70,87,88].

The diabetic kidney phenotype, with diabetic kidney disease and albuminuric CKD, represents a subgroup in which inflammation, fibrosis, and metabolic stress converge are present. In these patients, overactivation of mineralocorticoid receptor pathways contributes to progressive cardiorenal remodeling. Recent trials involving non-steroidal mineralocorticoid receptor antagonists such as finerenone demonstrated significant reductions in renal and cardiovascular composite outcomes, supporting their role in attenuating inflammatory and profibrotic signaling beyond conventional hemodynamic effects. Nonetheless, their implementation may be limited by hyperkalemia risk, particularly in patients with advanced CKD or concomitant renin–angiotensin system inhibition, highlighting the need for careful patient selection and monitoring. Emerging evidence also supports a renoprotective role of GLP-1 receptor agonists. In patients with type 2 diabetes and chronic kidney disease, GLP-1-based therapies have been associated with reductions in albuminuria, slower decline in estimated glomerular filtration rate, and decreased risk of progression to kidney failure. These findings have been further strengthened by the recently reported FLOW trial, which demonstrated significant kidney protective effects of semaglutide in individuals with diabetic kidney disease [63,72,89,90,91].

Finally, the cardio–metabolic phenotype occupies an intermediate position characterized by simultaneous cardiac and metabolic dysfunction, often associated with obesity-related HFpEF, atrial remodeling, and impaired metabolic flexibility. In these individuals, multimodal interventions combining SGLT2 inhibitors, GLP-1 receptor agonists, and aggressive management of metabolic risk factors may provide synergistic benefits [62,68,92,93,94,95,96,97,98]. However, despite the increasingly recognized cardiovascular and renal benefits of GLP-1 receptor agonists, earlier meta-analyses reported neutral effects on heart failure hospitalization, suggesting that treatment effects may not be uniform across all phenotypes and could vary according to predominant biological mechanisms. Consequently, recent findings supporting obesity-related HFpEF should be interpreted within the context of evolving evidence rather than generalized as a class effect across all heart failure subtypes [99,100]. The coexistence of myocardial dysfunction and systemic metabolic abnormalities likely explains the substantial heterogeneity observed within this phenotype and underscores the need for integrated therapeutic strategies.

Notably, machine-learning phenotyping in HFpEF suggests that patients with the highest CKM burden (obesity/diabetes/CKD) may derive the greatest reduction in HF rehospitalization from SGLT2 inhibitors and may show signals of benefit from ARNI, reinforcing the rationale for phenotype-enriched implementation strategies [53].

Importantly, the progressive expansion of disease-modifying therapies raises practical considerations extending beyond biological efficacy. Combining multiple agents with complementary mechanisms may theoretically maximize organ protection; however, increasing therapeutic complexity may contribute to polypharmacy, reduced adherence, drug-to-drug interactions, and substantial economic burden. These challenges may become particularly relevant in elderly and multimorbid CKM populations, where treatment tolerability and implementation often differ substantially from clinical trial settings [12,74,101].

Taken together, these observations support a phenotype-guided therapeutic framework in which therapies with cross-organ protective effects are deployed according to dominant biological mechanisms rather than disease stage alone. Future precision medicine approaches should aim not only to identify biological responders but also to determine optimal sequencing and prioritization of therapies according to phenotype characteristics, comorbidity burden, and real-world feasibility. Such an approach may more effectively address the heterogeneity of CKM syndrome and improve long-term cardiorenal outcomes.

## 8. Conclusions

Cardiorenal–metabolic (CKM) syndrome represents a complex and dynamically evolving condition in which metabolic, renal, and cardiovascular dysfunction are tightly interconnected through a network of shared biological pathways. While the staging system proposed by the American Heart Association provides a valuable framework for risk stratification and population-level characterization, it remains inherently limited in capturing the mechanistic heterogeneity and multidirectional nature of disease progression.

In this context, a phenotype-oriented approach offers a more physiologically grounded perspective, recognizing that CKM syndrome is not a uniform trajectory but rather a spectrum of distinct yet overlapping pathophysiological states. The identification of dominant biological drivers, ranging from metabolic dysfunction and vascular stiffness to inflammation and hemodynamic congestion, enables a more precise interpretation of disease expression and progression across individuals.

Current epidemiological and pathophysiological assumptions support a paradigm shift from stage-based to mechanism-driven management, in which phenotypic characterization complements traditional risk stratification to guide therapeutic prioritization. Future research integrating longitudinal phenotyping, multimodal imaging, and biomarker profiling will be essential to validate the proposed approaches.

## Data Availability

No new data were created or analyzed in this study. Data sharing is not applicable to this article.

## Abbreviations

The following abbreviations are used in this manuscript:

CKM	Cardiorenal–metabolic
CKD	Chronic kidney disease
CVD	Cardiovascular disease
AHA	American Heart Association
BMI	Body mass index
RAAS	Renin–angiotensin–aldosterone system
FGF-23	Fibroblast growth factor-23
NO	Nitric oxide
ET-1	Endothelin-1
PREVENT	Predicting Risk of cardiovascular disease EVENTs (risk score)
UK Biobank	United Kingdom Biobank
HF	Heart failure
HFrEF	Heart failure with reduced ejection fraction
HFpEF	Heart failure with preserved ejection fraction
LVH	Left ventricular hypertrophy
DKD	Diabetic kidney disease
RPGN	Rapidly progressive glomerulonephritis
eGFR	Estimated glomerular filtration rate
SGLT2	Sodium–glucose cotransporter-2
SGLT2i	Sodium–glucose cotransporter-2 inhibitors
GLP-1	Glucagon-like peptide-1
GLP-1 RAs	Glucagon-like peptide-1 receptor agonists
MRA	Mineralocorticoid receptor antagonists
VGF	VGF nerve growth factor inducible protein
TLQP-21	VGF-derived peptide TLQP-21
CV	Cardiovascular
